# Depression and anxiety among people living with HIV on antiretroviral therapy: prevalence and correlates at the National Sexually Transmitted Diseases/AIDS programme, in Sri Lanka

**DOI:** 10.1186/s13104-025-07550-3

**Published:** 2025-11-21

**Authors:** Lahiru Yasas Akuratiyage, Pushpa De Silva, Kumari Nirupama Karunarathna, Geethani Samaraweera, Sithum Manjika, Pubudu Upekshana Chulasiri, Chathurie Uththarika Suraweera

**Affiliations:** 1 National Institute of Mental Health, Mulleriyawa New Town, Sri Lanka; 2 National STD/AIDS programme, No.29, De Saram place, Colombo 10, Sri Lanka; 3 Department of Community Medicine, Faculty of Medical Sciences, University of Sri Jayewardenepura, Nugegoda, Sri Lanka; 4 Anti Malaria Campaign, Colombo 05, Sri Lanka; 5 University Psychiatry Unit, National Hospital, Colombo, Sri Lanka

**Keywords:** HIV, Depression, Anxiety, Antiretroviral therapy (ART), Mental Health

## Abstract

**Objective:**

Depression and anxiety are common among people living with HIV (PLHIV), leading to poor quality of life and reduced adherence to antiretroviral therapy (ART). Evidence from Sri Lanka is limited. This study aimed to assess the prevalence and correlates of depression and anxiety among Sinhala speaking PLHIV receiving ART at the National STD/AIDS Control Programme, Colombo.

**Results:**

A cross sectional study of 320 PLHIV used the validated Sinhala Hospital Anxiety and Depression Scale (HADS-S) and diagnostic interviews. Anxiety prevalence was 27.5% and depression 33.4%. Univariate analyses showed significant associations with female gender (anxiety *p* = 0.009; depression *p* < 0.001), low income (anxiety *p* = 0.005; depression *p* < 0.001), semi-urban residence (anxiety *p* = 0.003; depression *p* = 0.001), sexual minority/transgender identity (anxiety *p* < 0.001), sexual dysfunction (anxiety *p* = 0.001; depression *p* < 0.001), poor ART adherence (anxiety *p* < 0.001), marital status (anxiety *p* = 0.016; depression *p* < 0.001), non-disclosure (anxiety *p* = 0.008; depression *p* < 0.001), and suicidality (both *p* < 0.001). In multivariate regression, poor ART adherence predicted anxiety (OR = 16.45, 95% CI 6.48–41.75). Predictors of depression were HIV awareness > 1 year (OR = 10.10, 95% CI 1.16–87.76), female gender (OR = 3.32, 95% CI 1.42–7.77), marital status (married vs other, OR = 4.95, 95% CI 1.89–12.98), and income ≤ Rs.50,000 (OR = 2.74, 95% CI 1.35–5.56). Anxiety and depression correlated moderately (r = 0.319, *p* < 0.001). Only 8.1% received antidepressants despite 21.3% reporting suicidality.

## Introduction

The global Human Immunodeficiency Virus (HIV) landscape has changed with antiretroviral therapy (ART), transforming HIV into a manageable chronic condition [1]. Prevalence varies worldwide-20% in South Africa, 0.22% in India [2]-while Sri Lanka maintains a low-level epidemic (< 0.1%) but lacks mental health research among people living with HIV (PLHIV) [2]. Despite improved ART adherence, only the third Joint United Nations Programme on HIV/AIDS (UNAIDS) 90–90–90 target (viral suppression) has been achieved in Sri Lanka; 20% remain undiagnosed out of an estimated 3,700 PLHIV [3]. Recent guidelines recommend routine depression screening, and psychiatric services are increasingly being incorporated into HIV clinics [4].

The Diagnostic and Statistical Manual of Mental Disorders, Fifth Edition (DSM-5), defines depression as having five or more symptoms lasting at least 2 weeks, including depressed mood or anhedonia. Anxiety entails persistent unease and avoidance.

Depression and HIV share a bidirectional link; depression increases the risk of HIV, and HIV can lead to depression through nervous system effects, ART side effects, stigma, and illness-related stress [5, 6]. Among PLHIV, depression affects 34%, and anxiety affects 21%, which is approximately three times higher than in the general population [7]. The only Sri Lankan study conducted in 2009 reported a 30.1% prevalence of depression but did not assess anxiety or psychosocial factors [8].

It can occur at any stage of HIV infection, with more severe cases in advanced disease [9]. If untreated, it results in poor ART adherence, low CD4 counts, high viral loads, and a reduced quality of life [10].

Risk factors include female gender, low socioeconomic status, substance use, weak social support, and neuroticism [11, 12]. Overlap with HIV symptoms complicate diagnosis. The Hospital Anxiety and Depression Scale(HADS), particularly the Sinhala version (HADS-S), validated in local outpatients, was used for this study because of its ability to assess both anxiety and depression while reducing somatic symptom overlap [13].

## Material and methods

This cross sectional study was conducted at National STD/AIDS Programme in Sri Lanka to evaluate the prevalence of depression and anxiety among PLHIV receiving ART and to examine associated factors. Using systematic sampling, the study team recruited 320 eligible participants (PLHIV aged 18 years or older, who were Sinhala-literate) based on sample size calculations that accounted for expected depression (31%) and anxiety (22.2%) prevalence rates, with a 95% confidence interval and a 5% margin of error. Exclusion criteria included cognitive impairments or communication difficulties. Data collection involved administering the Sinhala validated Hospital Anxiety and Depression Scale(HADS-S), a 14-item instrument with separate anxiety and depression subscales (scores 0–21 each while 0–7 indicate no anxiety or depression, while of 8–21 suggest the presence of anxiety or depression), along with sociodemographic questionnaires. Participants scoring above clinical thresholds were referred to the closest psychiatry unit for confirmation and further management. All data were collected confidentially and analyzed using SPSS version 21, employing descriptive statistics, chi-square tests, correlation analyses, and multiple regression. The study received ethical approval from the National Hospital Sri Lanka Ethics Review Committee, with written informed consent obtained from all participants. Strict confidentiality measures were implemented, including secure data storage and protection of participant identities. The study acknowledged the potential stigma surrounding mental health among PLHIV but emphasized the importance of addressing these challenges proactively. Its primary aim was to enhance mental health screening and strengthen ART adherence by ensuring the early identification of affected individuals and timely referral for care. The findings will be disseminated through scientific publications, with the goal of informing future strategies for integrating mental health services into HIV care.

## Results

The study involved 320 participants (75% male, with a mean age of 43.82 ± 13.25 years), comprising 38.1% who were under 34 years and 28.8% who were over 54 years. Most lived in semi-urban (46.9%) or rural (40.9%) areas. Sexual orientation was mainly heterosexual (70.6%), with 95% identifying as cisgender. The relationship status was as follows: 45.9% married, 24.4% single, and 29.7% had children. Education levels varied, with 41.6% of the population completing O/L exams. Employment was reported by 75.9% of respondents, mainly as skilled labourers (22.6%) or entrepreneurs (19.3%), with 53% earning less than Rs 50,000 per month. The housing status indicated that 70% of the participants lived in their own homes.

HIV disclosure(to a spouse or closest relative) took place in 78.1% of cases. 77.5% reported HIV-related sexual dysfunction (53.6% loss of libido, 21.8% vaginismus/dyspareunia, 18.1% erectile dysfunction). Partners' HIV status was positive (29.4%), negative (39.1%), or unknown (31.6%). Most participants (59.1%) were aware of their status for over three years. Mental health impacts included an increase in depression diagnoses (19.4% post-HIV vs 9.7% pre-HIV), with 38.7% of those depressed currently on medication. Suicide attempts or self-harm were reported by 21.3%, and 41.3% used psychoactive substances.

### Overall findings of anxiety and depression

The study found that 52.5% of participants (n = 320) had no anxiety or depression, while 47.5% experienced at least one condition—34.1% had either anxiety or depression, and 13.4% reported both(Table 1). Reliability testing demonstrated good internal consistency for both HADS-S domains (Anxiety α = 0.742; Depression α = 0.705), with excellent overall scale reliability (α = 0.769) which were slightly lower than those reported in the original English validation (typically 0.80–0.90). A significant moderate positive correlation emerged between anxiety and depression (r = 0.319, *p* < 0.001), confirming their frequent co-occurrence. Both conditions exhibited non-normal distributions (Anxiety skewness = 1.048; Depression skewness = 1.202), supporting the use of non-parametric statistical analysis.

**Table 1:** Overall prevalence of anxiety and depression among PLHIV (n = 320)

Mental health status	n	%
No anxiety or depression	168	52.5%
Either anxiety or depression(1 condition)	109	34.1%
Both anxiety and depression(both conditions)	43	13.4%
Total with anxiety and/or depression	152	47.5%

### Anxiety

The study found that 27.5% (88) of participants (n = 320) scored within the anxiety range (HADS-A 8–21), of which 9.1% (29) met the threshold for clinical anxiety (HADS-A 11–21). Common symptoms included tension (87.6%), restlessness (86.3%), and feelings of impending doom (65%). Anxiety prevalence was significantly higher among females compared to males (38.8% vs 23.8%; χ^2^ = 6.771, *p* = 0.009), transgender individuals compared to cisgender participants (68.8% vs 25.3%; χ^2^ = 14.374, *p* < 0.001), and homosexuals compared to heterosexuals (48.7% vs 20.8%; χ^2^ = 18.211, *p* < 0.001). Lower-income earners (< Rs.50,000) reported higher anxiety than those with higher income (34.5% vs 19.0%; χ^2^ = 8.050, *p* = 0.005). Clinical correlates included sexual dysfunction (75%; χ^2^ = 18.565, *p* = 0.001), poor ART adherence (83.3%; χ^2^ = 80.597, *p* < 0.001), and self-harm (55.9% vs 19.8%; χ^2^ = 34.889, *p* < 0.001). Residence type was also significant, with semi-urban participants showing the highest anxiety (34.7% vs urban 7.7% and rural 25.2%; χ^2^ = 11.889, *p* = 0.003). Non-disclosure of HIV status to spouse/relative was associated with higher anxiety (40.0% vs 24.0%; χ^2^ = 7.022, *p* = 0.008). Binary logistic regression analysis (overall classification accuracy 82.4%) identified poor ART adherence as the strongest predictor of anxiety (OR = 16.45, 95% CI 6.48–41.75, *p* < 0.001). Urban/semi-urban residence also increased risk (OR = 3.31, 95% CI 1.12–9.74, *p* = 0.03), while non-disclosure of HIV status to a spouse/relative was associated with a protective effect (OR = 0.25, 95% CI 0.09–0.71, *p* = 0.009)(Table 2). No significant associations were observed with age, education, employment, partner’s HIV status, duration of diagnosis, comorbidities, substance use, viral load, or WHO stage. The wide confidence intervals for some predictors indicate imprecision, most likely due to inadequate subgroup sizes.

**Table 2:** Regression analysis with anxiety and other various factors (n = 320)

Explanatory variable	Reference category	B	S.E	Wald	df	Sig	Exp(B)	95% CI Lower	95% CI Upper
Resident	Rural, Urban and semi urban	1.196	0.551	4.71	1	0.03	3.308	1.123	9.745
Sexual Orientation	Hetero and Bi Sexual, Homo Sexual	1.282	0.754	2.892	1	0.089	3.603	0.822	15.786
Income	> Rs.50,000, = < Rs.50,000	0.615	0.418	2.164	1	0.141	1.851	0.815	4.202
Type of accommodation	Own home, Other places	0.746	0.478	2.441	1	0.118	2.109	0.827	5.376
Reveal the test result to spouse/closest relative	Yes, No	− 1.37	0.521	6.92	1	0.009	0.254	0.092	0.705
Anti-retroviral medication adherence	Good Adherence, Poor and moderate Adherence	2.8	0.475	34.732	1	0.0	16.451	6.482	41.752
Gender	Male, Female	− 0.071	0.487	0.021	1	0.884	0.932	0.358	2.421
Gender Identity	Cisgender, Transgender	0.711	1.403	0.257	1	0.612	2.037	0.13	31.863
Constant		− 2.698	0.594	20.624	1	0.0	0.067		

### Depression

The study found that 38.7% (n = 124) of participants reported feeling less energetic, 53.8% (n = 171) experienced reduced enjoyment (including 30.0% [n = 95] with anhedonia), 52.2% (n = 166) showed less interest in self-care, and 30.6% (n = 97) reported a diminished sense of humour. Overall, 33.4% (n = 106) had depressive symptoms (HADS-D score 8–21), with 15.9% (n = 50) meeting criteria for clinical depression (HADS-D score 11–21), while 66.6% (n = 212) scored within the normal range.

Depression was significantly higher among females compared to males (66.3% vs. 22.5%; χ^2^ = 51.599, *p* < 0.001), and lower among urban dwellers (15.4%) than in semi-urban (43.3%) and rural (27.5%) residents (χ^2^ = 14.399, *p* = 0.001). Rates were higher among married individuals (44.2% vs. 24.3%; χ^2^ = 14.197, *p* < 0.001) and parents (42.0% vs. 22.3%; χ^2^ = 13.691, *p* < 0.001). Low-income earners had significantly greater rates (< Rs.50,000: 45.8% vs. > Rs.50,000: 19.8%; χ^2^ = 20.130, *p* < 0.001). Non-disclosure of HIV status was protective (12.9% vs. 39.2%; χ^2^ = 17.051, *p* < 0.001). Among those with anorgasmia, 75% had depression (χ^2^ = 26.082, *p* < 0.001); 55.9% of those with a suicidal or self-harm history (χ^2^ = 19.545, *p* < 0.001) and 42.0% of psychoactive substance users (χ^2^ = 15.088, *p* < 0.001) met depression criteria. Depression was lower among those aware of their HIV status for over three years (33.9%) compared with those diagnosed within 1–3 years (47.1%) or less than 1 year (16.4%; χ^2^ = 13.885, *p* = 0.001). Current mental health treatment was associated with higher depression prevalence (61.5% vs. 32.2%; χ^2^ = 4.808, *p* = 0.028), likely reflecting greater symptom severity and help-seeking behaviour.

Binary logistic regression identified five independent predictors of depression (Table 3): awareness of HIV diagnosis > 1 year (OR = 10.1, 95% CI 1.2–87.8, *p* = 0.036), previous depression treatment after HIV diagnosis (OR = 6.0, 95% CI 1.9–18.7, *p* = 0.002), being married (OR = 4.9, 95% CI 1.9–13.0, *p* = 0.001), female gender (OR = 3.3, 95% CI 1.4–7.8, *p* = 0.006), and income ≤ Rs.50,000 (OR = 2.7, 95% CI 1.4–5.6, *p* = 0.005). No significant associations were found with age, education, employment, sexual orientation, religion, housing type, or CD4 counts.

**Table 3:** Regression analysis with depression and other various factors (n = 320)

Explanatory variable	Reference category	B	S.E	Wald	df	Sig	Exp(B)	95% CI Lower	95% CI Upper
Gender	Male, Female	1.199	0.434	7.613	1	0.006	3.315	1.415	7.768
Resident	Rural, Urban and semi urban	− 0.039	0.388	0.01	1	0.921	0.962	0.449	2.059
Marital status	Other, Married	1.599	0.492	10.55	1	0.001	4.947	1.885	12.98
Income	> Rs.50,000, = < Rs.50,000	1.009	0.36	7.834	1	0.005	2.743	1.353	5.559
Reveal the test result to spouse/closest relative	No, Yes	0.14	0.558	0.063	1	0.802	1.15	0.385	3.431
Previously diagnosed/treated for depression after HIV diagnosis	No, Yes	1.79	0.58	9.52	1	0.002	5.992	1.921	18.683
Attempted suicide/self-harm following diagnosis	No, Yes	0.318	0.425	0.559	1	0.454	1.375	0.597	3.165
Currently using psychoactive substances	Yes, No	0.431	0.42	1.053	1	0.305	1.539	0.676	3.503
Duration of knowing HIV status	= < 1 Year, 1 or more Years	2.313	1.103	4.397	1	0.036	10.103	1.163	87.759
Anti-retroviral medication adherence	Good Adherence, Poor and moderate Adherence	0.28	0.419	0.449	1	0.503	1.324	0.583	3.007
Constant		− 5.435	1.159	21.975	1	0.0	0.004		

## Discussion

The study found that the participant profile predominantly male (75%) with a mean age of 43.8 years, aligns with global PLHIV demographics [2]. The high rate of self-reported heterosexuality (70.6%) contrasts with global trends, suggesting possible underreporting due to stigma or fear of disclosing sexual orientation [14]. Sexual dysfunction was highly prevalent (77.5%), consistent with global data on HIV and impaired sexual health [15]. Despite history of high suicidal ideation (21.3%), mental health service utilization was low (8.1%), revealing a significant gap in integrated care [16]. Substance use was reported by 41.3%, echoing global patterns of coping with HIV-related stress and stigma [17].

Socioeconomic trends reflected global findings, with most participants employed but earning low to moderate incomes, indicating HIV’s financial impact [18]. Sexual dysfunctions such as loss of libido and erectile dysfunction are common [19]. HIV status disclosure was relatively high (78.1%), surpassing global averages, though stigma still posed a barrier [20]. Most participants resided in semi-urban or rural areas (87.8%), pointing to disparities in healthcare access in resource-limited settings.

### Anxiety in PLHIV

The study found a 27.5% ((95% CI 22.6%–32.4%) prevalence of anxiety among PLHIV in Sri Lanka-lower than in sub-Saharan Africa and the Americas (40–60%), where stigma and socioeconomic stressors increase rates up to 50% [21]. Females experienced higher anxiety levels (38.8%) than males (23.8%) statistically significant difference (χ^2^ = 6.771, *p* = 0.009) reflecting gender-specific stressors such as stigma, fears of disclosure, and relationship difficulties. Low income (less than Rs.50,000) was significantly associated with anxiety (34.5% vs. 19.0%; χ^2^ = 8.050, *p* = 0.005), reinforcing the link between financial insecurity and poor mental health. Anxiety was more common in semi-urban areas, possibly due to limited access to care, although global data often associates rural residence with higher anxiety [22].

Anxiety levels were significantly higher among homosexual (48.7%; χ^2^ = 18.211, *p* < 0.001) and transgender (68.8%; χ^2^ = 14.374, *p* < 0.001) participants, reflecting vulnerabilities caused by marginalisation and minority stress. Sexual dysfunction, notably anorgasmia (75%), was strongly linked to anxiety, emphasizing HIV’s impact on emotional wellbeing [23]. Suicidal ideation and self-harm affected 55.9% of those with anxiety, aligning with global trends and underlining the necessity for targeted interventions [24].

Unlike previous studies, anxiety was not significantly linked to age, education, or employment, despite evidence that higher education can improve coping. Poor ART adherence (83.3%) among anxious individuals highlights the connection between untreated mental illness and substandard HIV care.

### Depression in PLHIV

The study reported a 33.4% prevalence of depression among PLHIV in Sri Lanka, aligning with global (~ 30.6%) and regional rates from India (47%) and Africa (43%) [25, 26]. Depression was significantly higher in females (66.3%) than males (22.5%), reflecting caregiving burdens and stigma [27]. Urban participants had the lowest depression rates (15.4%), likely due to better access to care [22]. Higher rates were observed among married individuals (44.2%) and parents (42.0%), indicating added stress from caregiving and relationship roles [25, 26].

Low income (< Rs. 50,000) was strongly linked with depression (45.8%), consistent with associations between financial insecurity and poor mental health in PLHIV [28]. Depression was lower among those who hadn’t disclosed their HIV status (12.9%), highlighting the psychological strain of post-disclosure stigma [29]. Sexual dysfunction, especially anorgasmia, was strongly associated with depression (75.0%), demonstrating HIV’s effect on intimacy and well-being [15]. Suicidal ideation and self-harm were observed in 55.9% of depressed individuals, emphasising the need for accessible mental health care [24].

Unlike previous research, no significant associations were identified between depression and age, education, sexual orientation, gender identity, or employment, indicating that HIV stigma might outweigh usual demographic risk factors.

### Comparison between domains and global/regional findings

Anxiety and depression among PLHIV in this study were influenced by gender, income, sexual orientation, and HIV status disclosure, with anxiety more strongly associated with poor ART adherence and limited access to mental health services, while depression was closely linked to low income. These findings align with global [21] and regional literature, which report high mental health burdens among PLHIV, particularly among women [27], sexual minorities, and economically disadvantaged groups. Like African and South Asian studies, gender and minority status were key determinants, emphasising the need for culturally and socioeconomically responsive integrated mental health services [30].

### Strengths and limitations

The study surveyed 320 PLHIV, providing a large and diverse sample that improves generalizability. Using the validated HADS, it found notable prevalence rates of anxiety (27.5%) and depression (33.4%). Statistical tests, including chi-square and Fisher’s Exact tests, confirmed significant links between mental health outcomes and factors such as gender, income, and sexual orientation. The scale showed strong reliability (Cronbach’s alpha: 0.742 for anxiety, 0.705 for depression), and a moderate positive correlation was observed between anxiety and depression (r = 0.319, *p* = 0.000).

The study’s reliance on the Sinhala-validated HADS tool excluded non-Sinhala-speaking participants, limiting ethnic representation and reducing generalizability to Sri Lanka’s minority groups, who may face distinct mental health challenges. Future research should employ multi-lingual or culturally adapted tools to capture the experiences of these populations. Conducted solely in Colombo, the findings may not extend to peripheral regions where psychosocial dynamics and healthcare access differ; multi-center or geographically diverse studies are warranted. The narrow focus on anxiety and depression excluded other important mental health conditions, such as substance use disorders, underscoring the need for broader assessments within integrated HIV care models. The finding that non-disclosure appeared “protective” must be interpreted cautiously, as it may reflect avoidance of external stigma at the cost of internalized stress; importantly, disclosure-related stigma may also have contributed to the unexpectedly high reporting of heterosexual orientation, warranting further exploration in future research. Additional limitations include possible inconsistencies from multiple data collection methods, participant fatigue, and the confounding influence of Sri Lanka’s economic crisis on mental health and care access. While the HADS tool showed acceptable overall reliability, some items performed less well, and the absence of qualitative data constrained insights into stigma, coping, and sexual dysfunction.

## Conclusion

The study highlights a high prevalence of anxiety (27.5%) and depression (33.4%) among PLHIV, stressing the urgent need for targeted mental health interventions. Key influencing factors included gender, income, sexual orientation, sexual dysfunction, suicidal ideation, and ART adherence. The findings also emphasise the complex role of stigma and HIV status disclosure in shaping mental health outcomes. These findings underscore the need for integrated mental health services and personalised support to improve quality of life and treatment adherence in PLHIV, with future research needed on long-term outcomes and key psychosocial factors to inform targeted interventions and policies.

## Data Availability

The datasets used and/or analysed during the current study are available from the corresponding author on reasonable request.

## Abbreviations

PLHIV: People living with HIV
ART: Antiretroviral therapy
UNAIDS: Joint United Nations Programme on HIV/AIDS
HADS: Hospital anxiety and depression scale
HADS-S: Sinhala version of the hospital anxiety and depression scale
DSM-5: Diagnostic and statistical manual of mental disorders, fifth edition
CNS: Central nervous system
SPSS: Statistical package for the social sciences
O/L: Ordinary level (Sri Lankan secondary education exam)
Rs.: Sri Lankan rupees
WHO: World Health Organization
CD4: Cluster of differentiation 4 (immune cell count used in HIV monitoring)
STD/AIDS: Sexually transmitted diseases/acquired immune deficiency syndrome
OR: Odds ratio
α (alpha): Cronbach’s alpha (measure of internal consistency/reliability)
r: Pearson correlation coefficient
*p*: P-value (statistical significance level)
B: Regression coefficient (log odds)
S.E.: Standard error
Wald: Wald chi-square test statistic
df: Degrees of freedom
Sig.: Significance (*p*-value)
Exp(B): Exponentiated coefficient (odds ratio)
95% CI Lower: Lower bound of the 95% confidence interval for the odds ratio
95% CI Upper: Upper bound of the 95% confidence interval for the odds ratio
